# 31ièmes Journées Franco-Belges de Pharmacochimie: Meeting Report

**DOI:** 10.3390/ph10040094

**Published:** 2017-12-04

**Authors:** Raphaël Frédérick, Lionel Pochet, Pascal De Tullio, François Dufrasne

**Affiliations:** 1 Medicinal Chemistry Research Group (CMFA), Louvain Drug Research Institute (LDRI), Université Catholique de Louvain, Woluwé-Saint-Lambert 1200, Belgium; raphael.frederick@uclouvain.be; 2 Department of Pharmacy, Namur Medicine & Drug Innovation Center (NAMEDIC), Namur Research Institute for Life Sciences (NARILIS), University of Namur, 61, Rue de Bruxelles, Namur B-5000, Belgium; lionel.pochet@unamur.be; 3 Center for Interdisciplinary Research on Medicines (CIRM), University of Liege, Liège 4000, Belgium; P.DeTullio@ulg.ac.be; 4 Laboratoire de Chimie Pharmaceutique Organique, Faculté de Pharmacie, Université Libre de Bruxelles Campus Plaine CP 205/5, Brussels 1050, Belgium

**Keywords:** medicinal chemistry, pharmacochemistry, pharmacology, screening, target discovery, target validation

## Abstract

The “Journées Franco-Belges de Pharmacochimie” is a recognized two-day annual meeting on Medicinal Chemistry that is renowned for the advanced science presented, conviviality, and outstanding opportunities for senior and young scientists to exchange knowledge. Abstracts of plenary lectures, oral communications, and posters presented during the meeting are collected in this report.

## 1. Aim and Scope of the Meeting

The “Journées Franco-Belges de Pharmacochimie” (JFB) is a widely recognized annual meeting on Medicinal Chemistry. This two-day symposium aims to promote exchanges between medicinal chemists, mainly from France and Belgium. It is renowned for the advanced science presented, conviviality, and outstanding opportunities for senior and young scientists to exchange knowledge.

The scientific program included one tutorial lecture and four plenary lectures by internationally recognized scientists. An important part of the program was devoted to open lectures (nine oral communications) giving the opportunity for young scientists to present their research. The themes discussed during the meeting were those generally encountered by medicinal chemists: organic synthesis, bioinformatics and computer-aided drug design, pharmacological tests and molecular biology. This year, the official language for the lectures was English.

## 2. Plenary Lecture

### Exploring Hit Identification Strategies for the Energy-Coupling Factor Transporters: Towards Novel Antibiotics (PL3)

HirschAnna K. H.^1^,^2^,^3^1Stratingh Institute for Chemistry, University of Groningen, Nijenborgh 7, 9747AG Groningen, The Netherlands2Department of Drug Design and Optimization, Helmholtz Institute for Pharmaceutical Research Saarland (HIPS)—Helmholtz Centre for Infection Research, 38124 Braunschweig, Germany3Department of Pharmacy, Medicinal Chemistry, Saarland University, 66123 Saarbrücken, Germany; anna.hirsch@helmholtz-hzi.de

The challenges associated with anti-infective drug-discovery programs can be tackled by adopting several established and unprecedented hit-identification strategies, such as dynamic combinatorial chemistry (Mondal, M., et al. *Chem. Soc. Rev.* **2015**, *44*, 2455–2488). This approach will be illustrated using one target enzyme from the methyl erythritol phosphate pathway. This pathway provides a rich source of drug targets, given that pathogens such as *Mycobacterium tuberculosis* and *Plasmodium falciparum* use this pathway for the biosynthesis of the essential isoprenoid precursors isopentenyl diphosphate (IPP) and dimethylallyl diphosphate (DMAPP), while humans exclusively utilise the alternative mevalonate pathway (Masini, T., et al. *J. Med. Chem.* **2014**, *57*, 9740–9763). Our target enzyme 1-deoxy-d-xylulose-5-phosphate synthase (DXS) catalyses the first and rate-limiting step of the non-mevalonate pathway. To facilitate the development of potent and selective inhibitors of DXS, we have explored ligand- (Reymond, J.-L., et al. *ACS Chem. Neurosci.* **2012**, *3*, 649–657) and structure-based virtual screening, phage display, dynamic combinatorial chemistry (Mondal, M., et al. *Chem. Soc. Rev.* **2015**, *44*, 2455–2488), and de novo fragment-based design (Masini, T., et al. *Chem. Sci.* **2014**, *5*, 3543–3551). The most promising hits display inhibitory potency in the low micromolar range and promising activities in cell-based assays against *P. falciparum* and even drug-resistant strains of *M. tuberculosis*. Further assays demonstrated their selectivity over mammalian TDP-dependent enzymes, their lack of cytotoxicity, and validated DXS as the intracellular target (Hirsch, A.K.H., et al. EP15160746.2).

**Acknowledgments:** This work was funded by the Netherlands Organisation for Scientific Research (VIDI and LIFT grants), the Dutch Ministry of Education, Culture and Science (Gravitation Program 024.001.035) and the Helmholtz-Association’s Initiative and Networking Fund. 

## 3. Oral Communications

### 3.1. Synthesis and Biological Evaluation of Prodrugs with Acetylcholinesterase Inhibition and 5-HT_4_ Receptor Agonist Activities Targeting Alzheimer’s Disease (OC1)

ToubletFrançois-Xavier*LalutJulienLecouteyCédricDavisAudreyRochaisChristopheDallemagnePatrickCentre d’Etudes et de Recherche sur le Médicament de Normandie (CERMN), Normandie Univ, UNCEAN, 14000 Caen, France*Correspondence: francois-xavier.toublet@unicaen.fr

Alzheimer’s disease is a poorly understood multifactorial neurodegenerative disease. One of the molecular origins of the disease is the formation of amyloid plaques caused by a hyperactivation of β-secretase, which leads to the formation of the β-amyloid peptide. On the other hand, the hyperphosphorylation of tau protein leads to the formation of neurofibrillary tangles and disaggregation of microtubules (Yun, H., et al. *Exp. Neurobiol.* **2011**, *20*, 159–168). Due to the molecular complexity of the disease, a significant number of therapeutic targets have been investigated. However, the market counts few molecules with moderate activity, mostly acetylcholinesterase (AChE) inhibitors. Among them, rivastigmine is a covalent reversible inhibitor of AChE.

In this context, a new concept is studied: Multi-Target Directed Ligands (MTDLs), describing a drug with several therapeutic targets of interest to treat a disease. Donecopride, a MTDL, was synthesized in our laboratory (Lecoutey, C., et al. *Proc. Natl. Acad. Sci. USA* **2014**, *111*, E3825–E3830): this molecule could simultaneously inhibit AChE and activate serotonergic receptor 5-HT_4_ (5-HT_4_R), leading to promising procognitive properties in several animal models.

In our project, we have designed and synthesized prodrugs as structural analogs between donecopride and rivastigmine. These drugs possess a carbamate group, and therefore, could be AChE covalent inhibitors. This reversible AChE inhibition will ultimately release the corresponding phenolic derivative, which should be active on 5-HT_4_R.


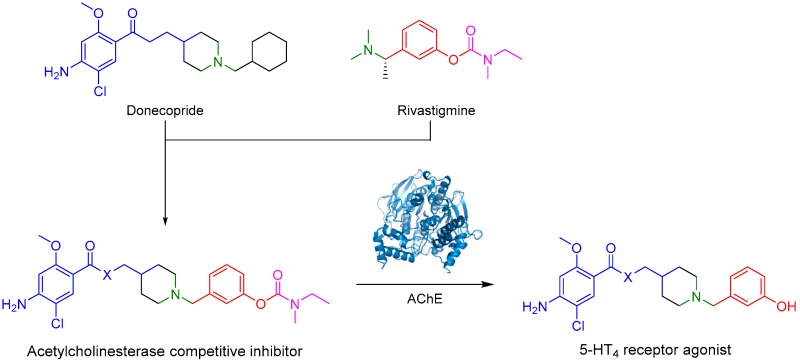


### 3.2. Synthesis of Original Serotonergic Multi-Target Directed Ligands—The Triad Program (OC3)

HatatBérénice^1^,^2^*LecouteyCédric^1^YahiaouiSamir^1^DavisAudrey^1^FreretThomas^3^BoulouardMichel^3^ClaeysenSylvie^2^RochaisChristophe^1^DallemagnePatrick^1^1Centre d’Etudes et de Recherche sur le Médicament de Normandie (CERMN)—UPRES EA 4258—FR CNRS INC3M—SFICORE, Université de Caen Normandie, UFR des Sciences Pharmaceutiques—Bd Becquerel, F-14032 Caen, France2CNRS, UMR-5203, Institut de Génomique Fonctionelle, F-34000 Montpellier, France3Unité COMETE UMR-S 1075 INSERM, 2 rue des Rochambelles, F-14032 Caen, France*Correspondence: berenice.hatat@unicaen.fr

Targeting more than one molecular cause implied in the pathogenesis of Alzheimer’s disease (AD) with a sole drug is considered a promising challenge, because it could address the failures that recently occurred during clinical trials. Within this framework, we recently reported the design of donecopride, a pleiotropic agent that both displays acetylcholinesterase (AChE) inhibition and 5-HT_4_R agonist activity (Lecoutey, C., et al. *Proc. Natl. Acad. Sci. USA* **2014**, *111*, E3825–E3830). Based on its procognitive and antiamnesiant in vivo properties, donecopride is currently under preclinical investigation. A pharmacomodulation study allowed to establish the structure–activity relationships in the donecopride series, and to enlarge the latter with some other potent ligands (Rochais, C., et al. *J. Med. Chem.* **2015**, *58*, 3172–3187). Taking into account the clinical interest of idalopirdine, a 5-HT_6_R antagonist, in Alzheimer’s disease (AD) treatment, we undertook a new program aiming at designing novel Multi-Target Directed Ligands targeting both, but selectively, AChE 5-HT_4_ and 5-HT_6_ receptors.

Considering the pharmacophores established for each of the three targets, we performed the synthesis of numerous donecopride derivatives. Among them, some dual compounds both exhibited in vitro 5-HT_4_R agonist and 5-HT_6_R antagonist activities, and displayed an in vivo procognitive effect. Pursuing the pharmacomodulation of these compounds led to first pluripotent derivatives with in vitro submicromolar activities towards the three designated targets.

The TRIAD program, funded by the Ligue Européenne contre la Maladie d’Alzheimer and the French Fondation Plan Alzheimer, recently led to some novel promising agents with in vitro prerequisites for further in vivo evaluation in AD experimental models.

### 3.3. Fragment-Based Drug Discovery Targeting Inhibitor of Apoptosis Proteins (OC4)

DenisCamille^1^*MarekhaBogdan^1^HedirSiham^2^LegayRémi^1^AbeilardEdwige^2^BrotinEmilie^2^PoulainLaurent^2^BureauRonan^1^Voisin-ChiretAnne Sophie^1^1Centre d’Etudes et de Recherche sur le Médicament de Normandie (CERMN), Normandie University, UNICAEN, 14032 Caen, France2Normandie Univ, UNICAEN, INSERM U10186 “ANTICIPE” Axe 2 BioTICLA, Centre François Baclesse, 4000 Caen, France*Correspondence: camille.denis@unicaen.fr

Protein–protein interactions (PPIs) control many important physiological processes within human cells. 

A hallmark of cancers is the evasion of apoptosis, which is often associated with the upregulation of the anti-apoptotic members of the Bcl-2 family of proteins. The Bcl-2 protein family comprise pro-survival and pro-apoptotic members. Interactions among these groups, involving binding of the BH3 domain of the pro-apoptotic members to a groove on the surface of the pro-survival proteins, control commitment to apoptosis. In many cancers, the balance between the pro- and anti-apoptotic Bcl-2 family members is tipped towards survival. Drugs inhibiting the pro-survival activity of Bcl-2 proteins to restore cell death may therefore be valuable as cancer therapeutics.

Even though potent and selective inhibitors of Bcl-2 and Bcl-x_L_ have been developed, it is not enough to reestablish apoptosis. Mcl-1 protein has been shown to mediate resistance to chemotherapeutics, which is why the discovery of dual Mcl-1/Bcl-x_L_ inhibitors could play an important role in the cancer treatment.

Our laboratory synthesized a selective inhibitor of Mcl-1 protein called Pyridoclax (Gloaguen, C., et al. *J. Med. Chem.* **2015**, *58*, 1644–1668). Starting from the structure of this lead, fragments have been designed and synthesized with the objective to target Mcl-1. By now, the different ways to achieve a dual action on Mcl-1 and Bcl-x_L_ will be investigated.


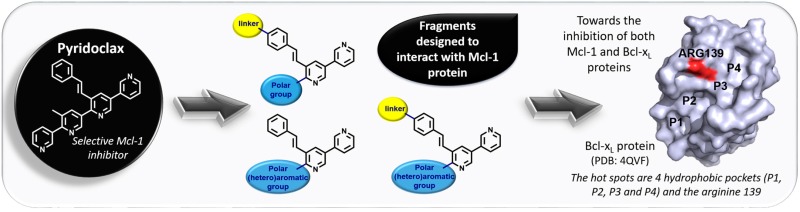


## 4. Posters

### 4.1. Structure Characterization of TMEM45A, a Protein Involved in the Resistance to Chemotherapy (P05)

MasoThomas Dal^1^*MarxSébastien^1^LeCalvéBenjamin^2^MichielsCarine^2^WoutersJohan^1^1Department of Chemistry, Laboratoire de Chimie Biologique Structurale (CBS), Namur Medicine and Drug Innovation Center (Namedic), Namur Research Institute for Life Sciences (NARILIS), University of Namur (UNamur), Namur B-5000, Belgium2Department of Biology, Unité de Recherche en Biologie Cellulaire (URBC), Namur Research Institute for Life Sciences (NARILIS), University of Namur (UNamur), Namur B-5000, Belgium*Correspondence: thomas.dalmaso@unamur.be

Cancer is the leading cause of death worldwide. The development of therapy resistance continues to be a major problem in the treatment of patients with cancer. Multiple processes influence tumor response to therapies. Treatment failure has been identified as one of the four major issues in cancer research. Recently, we developed new cellular models to demonstrate that tumor microenvironments, and especially hypoxia, promote tumor growth and resistance to treatment. In this light, we have identified the protein TMEM45A. Its expression is increased under hypoxic conditions, and when overexpressed, it shows anti-apoptotic properties and induces the resistance of cancer cells to chemotherapeutic agents. Inversely, the silencing of TMEM45A in hepatocellular carcinoma cells (HepG2) incubated in presence of etoposide under hypoxic conditions results in their sensitization to cell death induced by the anticancer agent (Rebucci, M., et al. *Biochem. Pharmacol.* **2013**, *85*, 1219–1226; Flamant, L., et al. *BMC Cancer* **2012**, *12*, 391; Hayez, A., et al. *Exp. Dermatol.* **2014**, *23*, 339–344; Cosse, J.P., et al. *Anti-Cancer Agents Med. Chem*. **2008**, *8*, 790–797).

This work focuses on the structural study (protein topology and 3D structure) of the membrane protein TMEM45A. Our first results evidenced a new isoform of the protein that remains to be further characterized. This seems to correspond to the major biological isoform of TMEM45A, at least in cancer cells. 


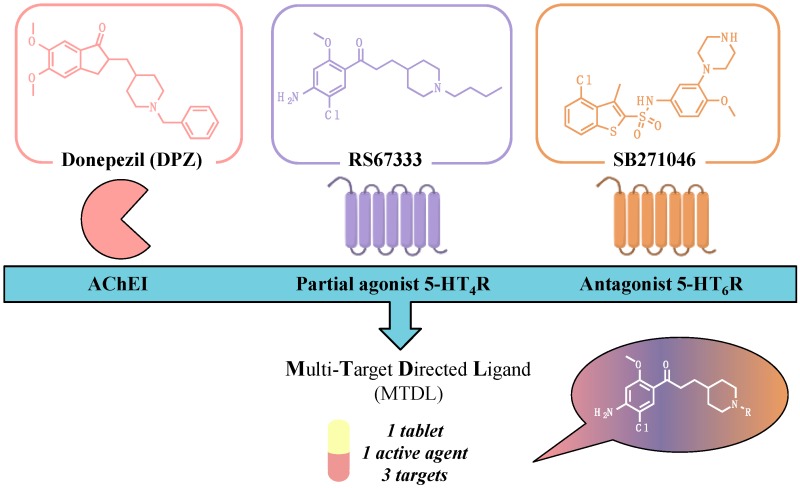


In order to study the membrane topology of this protein, we will use molecular biology techniques and modeling tools to create or validate 3D models for TMEM4A. Overexpression, purification, and crystallization (in lipidic cubic phase) (Ostermeier, C., et al. *Curr. Opin. Struct. Biol*. **1997**, *7*, 697–701) of the protein will also be carried out in eukaryotic cells and bacteria, to characterize the structure. Structural information will allow virtual screening of a chemical library and identification of potential ligands, as well as new insights on TMEM45A function and its involvement in the resistance mechanism to apoptosis induced by etoposide.

**Acknowledgments:** Author thanks URPHYM (Unité de Recherche en Physiologie Moléculaire). This work is supported by FRIA Grant. Thomas Dal Maso is grateful for financial support from the Fonds pour la Recherche Scientifique (FNRS).

### 4.2. Asymmetric Synthesis of Rhodotorulic Acid Analogues with Potential Siderophore Properties (P06)

GarnerinTimothée*Dassonville-KlimptAlexandraLohouElodieSonnetPascalLaboratoire de Glycochimie des Antimicrobiens et des Agroressouces (LG2A), UMR CNRS 7378, Université de Picardie Jules Verne, UFR de pharmacie, 1 rue des Louvels, F-80037 Amiens CEDEX 01, France*Correspondence: timothee.garnerin@etud.u-picardie.fr

Antibiotic resistance is an emerging disease and a real problem of health. Resistance of Gram negative bacteria, such as *Acinetobacter baumannii* and *Escherichia coli*, to conventional antibiotics, leads to therapeutic failure and requires new antibiotherapies. The use of the iron transport systems is one of the most promising strategies to overcome this resistance phenomenon. These specific routes of entry, essential for the survival of the microorganisms, allow ferric siderophore complexes to carry iron within the bacteria.

These systems allow the introduction of antibacterial agents (antibiotic–siderophore conjugates) or toxic complexes (gallium complexes) into the bacteria to kill them. Rhodotorulic acid (RA) is a siderophore transported by TonBox dependant Fhu receptors. These kinds of receptors are expressed by *Acinetobacter baumannii* and *Escherichia coli*. RA is dioxopiperazine iron chelator with hydroxamate as iron ligands and two asymmetric centers (*S*,*S* configuration). This spatial orientation is essential for the Fhu receptors recognition.

We have previously reported the asymmetric synthesis of 3-substituted 2-oxopiperazines. Herein, we present an original and a convergent strategy to synthesize RA and corresponding 3,6-disubstituted analogues. Siderophore-like tests and measurement of the complexing strength of these compounds will be carried out.


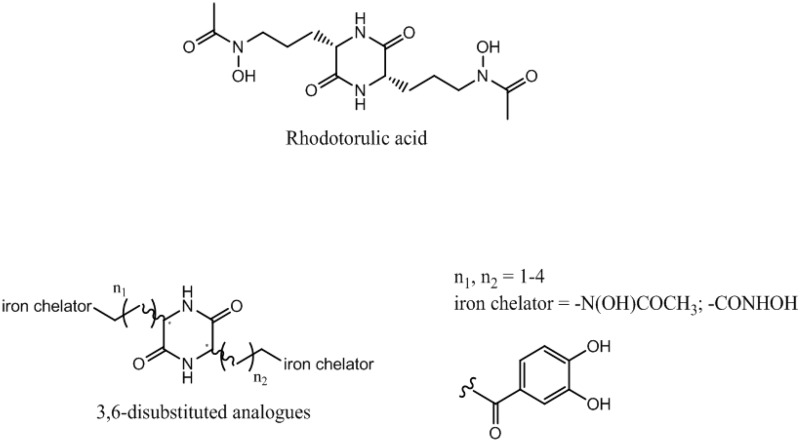


### 4.3. Design and Synthesis of 4-Aminoalcoholquinoline–Siderophore Conjugates (P07)

JourdanJean-PierreDassonville-KlimptAlexandra*MullieCatherineLohouElodieSonnetPascalLaboratoire de Glycochimie des Antimicrobiens et des Agroressouces (LG2A), UMR CNRS 7378, Université de Picardie Jules Verne, UFR de pharmacie, 1 rue des Louvels, F-80037 Amiens CEDEX 01, France*Correspondence: alexandra.dassonville@u-picardie.fr

The dissemination of multidrug-resistant bacteria has reduced the therapeutic efficacy of antibiotic drugs, and is now part of major human healthcare emergencies. Among the bacteria ESKAPE, Gram negative pathogens such as *Pseudomonas aeruginosa* or *Acinetobacter baumannii* are very virulent. 

Bacteria require iron for many vital functions. However, in the oxidative atmosphere, iron exists as insoluble salts which makes its bacterial assimilation hard. To overcome iron deprivation, pathogens use small iron chelators named siderophores, which transfer iron within bacteria by specific receptors. Siderophores are classified into four main families: hydroxamates, catecholates, carboxylates, and phenolates. Previous studies have shown that the motif hydroxypyridinone is recognized by the same kind of transporters than catechol groups. These siderophores moieties can be conjugated with antibiotic (antibiotic–siderophore conjugates) (Budzikiewicz, H., et al. *Curr. Top. Med. Chem.* **2001**, *1*, 73–82) or form toxic complexes (gallium complexes) to fight resistant Gram negative bacteria (Kelson, A.B., et al. *Curr. Opin. Pharmacol.* **2013**, *13*, 707–716). 


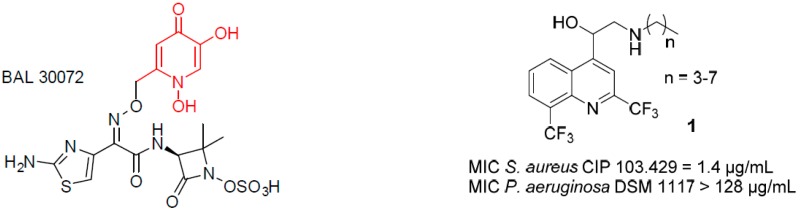


A 1,3-dihydroxypyridin-4-one as chelating group was recently combined to a monosulfactam, to lead to conjugate BAL30072, which is active against *P. aeruginosa* and *A. baumannii* multidrug-resistant strains (Page, M.G.P., et al. *Antimicrob. Agents Chemother*. **2010**, *54*, 2291–2302). In previous research, we have shown that aminoquinolinemethanols **1** (AQMs), such as mefloquine analogues, possess an antibacterial activity only against Gram positive bacteria (Jonet, A., et al. *J. Antibiot.* **2013**, *66*, 683–686). 


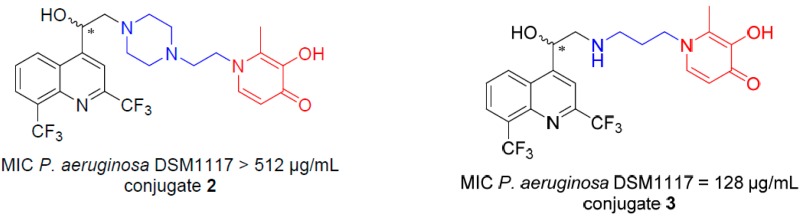


Inspired by the Trojan horse strategy, we grafted hydroxypyridinone group to the AQM moiety with different linkers, to obtain conjugates **2** and **3**. Conjugate **2** is not active against *P. aeruginosa* DSM 1117 while conjugate **3** possesses a significant activity (MIC 128 µg/mL).

### 4.4. Development of Multifunctional Diamine AGE/ALE Inhibitors with Potential Therapeutic Properties against Alzheimer's Disease (P11)

LohouElodie^1^*André SasakiN.^1^BoullierAgnès^2^,^3^,^4^SonnetPascal^1^1Laboratoire de Glycochimie des Antimicrobiens et des Agroressouces (LG2A), UMR CNRS 7378, Université de Picardie Jules Verne, UFR de pharmacie, 1 rue des Louvels, F-8003 Amiens CEDEX 01, France2Université de Picardie Jules Verne, UFR de Médecine, 1 Rue des Louvels, F-80037 Amiens CEDEX 01, France3INSERM U1088, Centre Universitaire de Recherche en Santé (CURS), Avenue René Laënnec—Salouel, F-80054 Amiens CEDEX 01, France4CHU Amiens Picardie, Avenue René Laënnec—Salouel, F-80054 Amiens CEDEX 01, France*Correspondence: elodie.lohou@u-picardie.fr

During the sugar glycoxidation and lipid peroxidation of polyunsaturated fatty acids induced by oxidative stress exacerbation, reactive carbonyl species (RCS) are endogenously formed and react with amino groups of tissue proteins to give AGE (Advanced Glycation Endproducts) and ALE (Advanced Lipid peroxidation Endproducts). In Alzheimer’s disease (AD), extensive AGE/ALE accumulation has been reported in extracellular amyloid *β* (A*β*) plaques and intracellular tau-associated neurofibrillary tangles (NFT). Indeed, a critical imbalance between cerebral reactive oxygen species (ROS) production and endogenous antioxidant capacities associated with biometal dyshomeostasis has been suggested to be a driving force for AD onset and progression. A*β*-oligomers induce oxidative stress, whereas transition metals (Zn^2+^, Cu^2+^ and Fe^3+^) stimulate A*β* aggregation and APP (amyloid precursor protein) processing. Consequently, RCS accumulation takes part in the vicious downward redox amyloid spiral leading to neurodegeneration (Butterfield, D.A., et al. *Trends Mol. Med.* **2001**, *7*, 548–554; Tiiman, A., et al. *Neurochem. Int.* **2013**, *62*, 367–378). AGE/ALE contribute to AD pathogenesis through three main mechanisms (Krautwald, M., et al. *Gerontol.* **2010**, *45*, 744–751). First, glycated A*β* cross-linking promotion accelerates its deposition and its protease resistance. Secondly, AGE/ALE formation not only accelerates tau hyperphosphorylation, disturbs the neuronal membrane depolarization process and the glucose transport, but also exacerbates glutamate-mediated excitotoxicity. Thirdly, AGE promote, via their receptors, RAGE oxidative stress and inflammation, as well as cell apoptosis. 

Taking into account the multifactorial pathogenesis of AD, we designed new multifunctional drugs that are simultaneously able to trap RCS (primary vicinal diamine function) as well as ROS and biometals (phenolic acid or hydroxypyridinone (HOPO) moiety) (Lohou, E., et al. *Eur. J. Med. Chem.* **2016**, *122*, 702–722). In the poster, synthesis of these new promising hybrid AGE/ALE inhibitors and evaluation of their physicochemical and biological properties (carbonyl trapping capacity, antioxidant activity, Cu^2+^-chelating capacity, cytotoxicity, and protective effect against in vitro MGO-induced apoptosis in the model AD cell-line PC12) will be described.


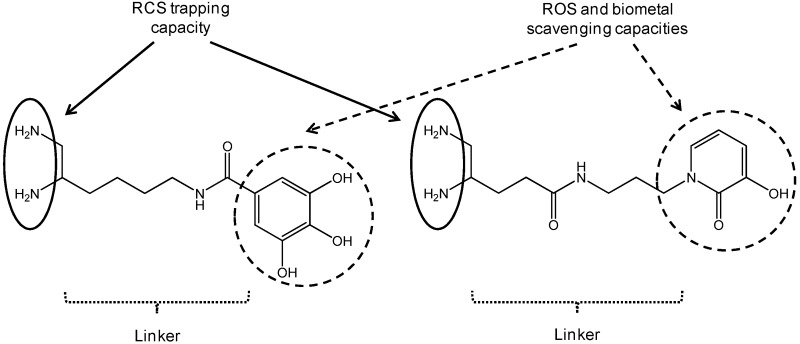


### 4.5. Design, Synthesis, and Biological Evaluation of New Fluoro-Indazole Derivatives as Potential PET Radiotracers for Brain 5-HT_4_ Receptors (P12)

MangeantReynald^1^,^2^*LamBao Vy^3^StiebingSilvia^1^,^2^CaillyThomas^1^,^2^DavisAudrey^1^,^2^FosseyChristine^1^,^2^FabisFrédéric^1^,^2^CollotValérie^1^,^2^1UFR Santé, Faculté des Sciences Pharmaceutiques, Université de Caen Normandie, 14032 Caen, France2Centre d’Etudes et de Recherche sur le Médicament de Normandie (CERMN), Normandie Univ, UNICAEN, 14000 Caen, France3Université Lille INSERM, Institut Pasteur de Lille, U1177-F-5900 Lille, France*Correspondence: reynald.mangeant@unicaen.fr

Since its discovery in 1988, the serotonin 4 receptor subtype (5-HT_4_R) has emerged as a promising target for drug discovery and development resulting from its implications in cognition, learning, and memory processes, and many neuropsychiatric disorders such as Alzheimer’s disease, anxiety, depression, or anorexia nervosa (Bockaert, J., et al. *Neuropharmacology* **2008**, *55*, 922–931). Thus, discovery of active 5-HT_4_R agonists and antagonists remains a continuing interest in clinical research. 


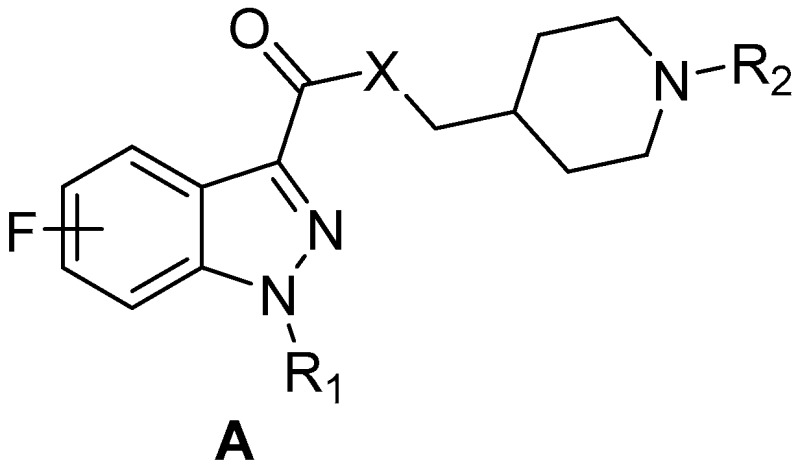


To this end, positron emission tomography (PET) (Marner, L., et al. *Neuroimage* **2010**, *50*, 855–861), (Caillé, F., et al. *Bioorg. Med. Chem. Lett.* **2013**, *23*, 6243–6247) coupled with effective radioligands constitutes valuable tool, both in clinical studies and drug discovery programs. Based on previous works in CERMN (Lam, B.V., et al. *Chem. Eur. J*. **2016**, *22*, 4440–4446), we aimed to develop new fluorinated indazole derivatives as potential brain 5-HT_4_R PET tracers.

In order to get more convergent and straightforward access to fluorinated analogues, we have established a simple methodology allowing selective functionalization of fluoroindazoles at position 3, and providing polyfunctional indazoles within a minimum of steps. 


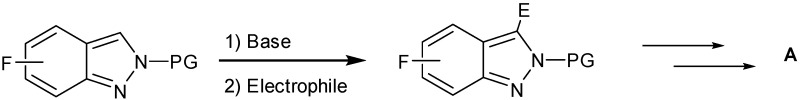


New indazole derivatives have been evaluated and four exhibiting nanomolar binding affinities for 5-HT_4_R could be radiolabeled then evaluated in PET imaging for brain 5-HT_4_R imaging.

### 4.6. Design and Synthesis of Novel Ruthenium(II) and Osmium(II) Complexes with Potential Antitumor Activity (P13)

MarloyeMickaël^1^*BergerGilles^1^IngelsAude^2^MeyerFranck^1^GelbckeMichel^1^MathieuVéronique^2^DufrasneFrançois^1^1Microbiology, Bioorganic & Macromolecular Chemistry, Faculté de Pharmacie, Université libre de Bruxelles, ULB, Boulevard du Triomphe, 1050 Brussels, Belgium2Laboratoire de Toxicologie, Faculté de Pharmacie, Université libre de Bruxelles (ULB, Boulevard du Triomphe, 1050 Brussels, Belgium*Correspondence: Mickael.Marloye@ulb.ac.be

Growing interest in the cytotoxic properties of piano-stool ruthenium(II) and osmium(II) arene complexes has developed over the past few years. Despite structural similarity, Ru and Os analogues come with different chemical, physical, and biological properties. (Morris; R.E.; et al. *J. Med. Chem.* **2001**, *44*, 3616–3621; Nowak-sliwinska, P., et al. *ChemMedChem* **2015**, *10*, 1539–1547).


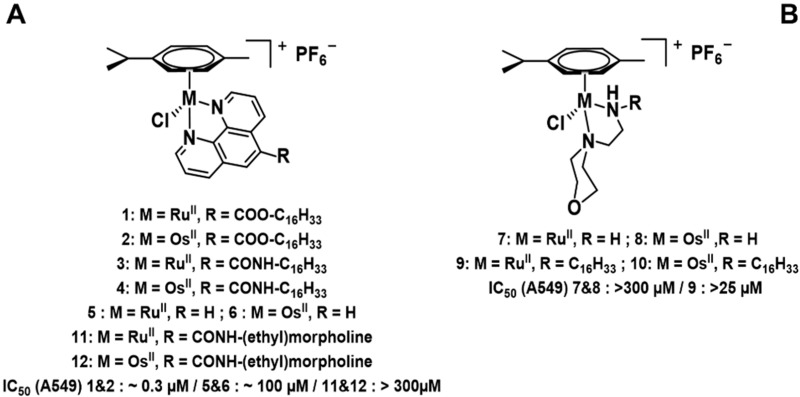


Novel Ru^II^ and Os^II^ half-sandwich complexes were synthesized from [(M(*p*-cymene)Cl_2_]_2_ (where M = Os^II^ or Ru^II^), using morpholine and phenanthroline derivatives as *N*,*N* bidentate donors. The in vitro antiproliferative effects were first assessed by means of MTT assays on a panel of six cancerous cell lines (A459, MCF-7, SKMEL-28, B16F10, HS638, U373). The ligands were further modified by adding long alkyl chains (C_16_) to help crossing membranes and enhance cellular accumulation.

C_16_-modified Ru^II^ and Os^II^ phenanthroline complexes showed promising results with IC_50_ values below 1 µM, and thus, potentially more effective than cisplatin, whereas the parent phenanthroline complexes [M(*p*-cymene)(phen)Cl]PF_6_ have very limited antiproliferative activity (Habtemariam, A., et al. *J. Med. Chem*. **2006**, *49*, 6858–6868). Therefore, following our strategy for increasing the cellular accumulation, a marked increase in the antiproliferative potency (100–1000 fold) was obtained. Cellular uptake experiments by ICP-MS will reveal the putative increase in the intracellular concentrations of the metal complexes. 

The morpholino complexes were found to be inactive with IC_50_ values over 300 µM, despite the presence of the C_16_ alkyl chain. Single crystals of these complexes were obtained for the determination of their solid-state structures, together with an NMR study in solution.


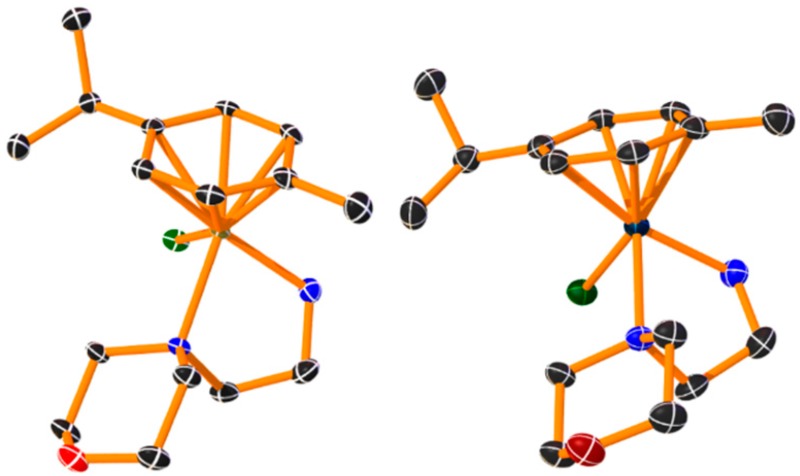


Further biological evaluation of these promising series of Os^II^ and Ru^II^ compounds is ongoing, using quantitative video microscopy, and apoptosis rate and cell cycle analysis. The stability (exchange reactions of chloride with water and nucleophiles) of the complexes will also be investigated.

### 4.7. Study of the Inhibition of SerB2 (Mycobacterium tuberculosis Phosphoserine Phosphatase) (P14)

PiersonElise^1^HaufroidMarie^1^*SinghRamandeep^2^WoutersJohan^1^1Laboratoire de Chimie Biologique Structurale (CBS), Namur Medicine and Drug Innovation Center (Namedic), Namur Research Institute for Life Sciences (NARILIS), University of Namur (UNamur), 5000 Namur, Belgium2Vaccine and Infectious Disease Reasearch Centre, Translational Health Science and Technology Institute, Gurgaon 122016, Haryana, India*Correspondence: marie.haufroid@unamur.be

Drug-resistant tuberculosis, caused by a strain of *Mycobacterium tuberculosis* resistant to first and second-line drugs, is a major global health threat (Sarkar, S., et al. *J. Pharm. Pharm. Sci.* **2011**, *14,* 148–161; Green, K., et al. *Front. Microbiol.* **2013**, *4*, 208; Da Silva, P.D.A., et al. *J. Antimicrob. Chemother.* **2011**, *66*, 1417–1430). Treatment options being limited, the search for new anti-tuberculosis targets and drugs is therefore needed. SerB2 is known to be a virulence factor of *M. tuberculosis* [Shree, S., et al., *Cell. Mol. Life Sci.* **2016**, *73*, 3401–3417) and to be essential for bacteria survival, which makes this enzyme a promising therapeutic target for tuberculosis treatment (Arora, G., et al. *J. Biol. Chem.* **2014**, *289*, 25149–25165).

SerB2 is secreted into the cytoplasm of THP-1 macrophages infected by *M. tuberculosis.* Through its phosphatase activity, the enzyme dephosphorylates definite proteins and transcription factors, which causes microtubule rearrangement within the macrophage and deactivates the immune system of the host. SerB2 then assists the bacteria in immune invasion and evasion (Sharma, A.K., et al. *Ind. J. Microbiol.* **2017**, *57*, 1–10; Yadav, G.P., et al. *PLoS ONE* **2014**, *9*, 1–24).


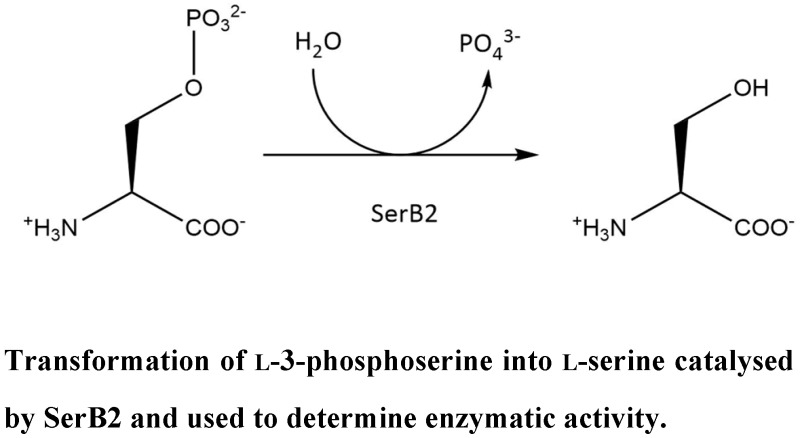


The objective of this work is to inhibit SerB2, with the overall aim of reducing *M. tuberculosis* virulence. The starting point was to express and purify SerB2. Then, new inhibitors were identified through an enzymatic activity assay using colorimetric phosphate determination. To achieve this, a screening of previously selected small molecules of the namedic’s library was performed. This experiment led to the identification of three promising inhibitors that are harmine derivatives. Clofazimine potent inhibitory effect was also confirmed. The inhibition type of the scaffolds will be assessed by further kinetic studies, and other derivatives will be synthesized and characterized. 

**Acknowledgments:** This work was funded by the University of Namur. The authors thank members of namedic who generated namedic library, in particular Prof B. Masereel and L. Pochet.

### 4.8. Synthesis of Novel Racemic and Enantiopure Antimalarial Agents with Fluorene Core (P16)

ScheinerJérémy^1^*Dassonville-KlimptAlexandra^1^Demailly-MullieCatherine^1^TaudonNicolas^2^LohouElodie^1^SonnetPascal^1^1Laboratoire de Glycochimie des Antimicrobiens et des Agroressouces (LG2A), UMR CNRS 7378, Université de Picardie Jules Verne, UFR de pharmacie, 1 rue des Louvels, F-80037 Amiens CEDEX 01, France2Institut de recherche biomédicale des armées (IRBA), RD 19, 91220 Brétigny-sur-Orge, France*Correspondence: jeremy.schneider@etud.u-picardie.fr

Malaria is a neglected tropical disease that remains a leading cause of morbidity and mortality among the world’s poorest populations. More than 100 tropical and sub-tropical countries are endemic for this infectious disease. Pregnant women and children are the most sensitive to this infection, and in 2015, 429,000 people died. Among the five species of *Plasmodium* responsible for human malaria, *P. falciparum* is the parasite which causes the most serious form of the disease. More recent efforts focused on the development of antimalarial vaccines and since 2001, World Health Organization (WHO) recommends artemisinin-based combination therapies (ACTs). In drug resistance areas, several antimalarial drugs, such as aminoalcohol-aryl (mefloquine (MQ), lumefantrine (LM)), are currently used in combination with artemisinin derivatives. However, the emergence of multidrug-resistant parasites decreases efficacy of ACTs. Thus, the design of new active compounds on *Plasmodium*-resistant strains is urgent. 

We have previously developed an asymmetric synthesis to prepare 4-aminoalcohol-quinoline enantiomers (AQ) as MQ analogs. They were active at the nanomolar range against 3D7 (chloroquine-sensitive) and W2 (chloroquine-resistant) *P. falciparum* strains. Interestingly, (*S*)-enantiomers displayed an activity increased by 2 to 15-fold as compared to their (*R*)-counterparts. During the *Plasmodium* intra-erythrocytic asexual stages, hemozoin formation and the oxidative and glutathione-dependent degradation of heme are inhibited by these aminoalcohol-aryls (MQ, LM). Currently, their mechanisms of actions are not totally clear, and remain to be explored.

In continuation of our work, we are interested in studying the change of heterocycle (fluorene vs quinoline) on the antimalarial activity. We focus on the design and the preparation of novel racemic and enantiopure aminoalcohol-fluorene derivatives (AFM) as LM analogs. The evaluation of their antiplasmodial activity against *P. falciparum*, and their corresponding cytotoxicity, is in progress.

**Acknowledgments:** J.S. was the recipient of a grant from DGA (Direction Générale de l’Armement, Ministère de la Défense, France) and Région Picardie.

## 5. Conclusions

At the end of the meeting, the following prizes were awarded:

- Prize for the best oral communication: C. Denis of the University of Caen (France) for her talk “Fragment-Based Drug Discovery targeting inhibitor of apoptosis proteins”
- Prize for the best poster presentation: M. Haufroid of the University of Namur (Belgium) for her poster “Study of the inhibition of SerB2 (*Mycobacterium tuberculosis* phosphoserine phosphatase)”.

In 2018, the “32ièmes Journées Franco-Belges de Pharmacochimie” will be held in France (Asnelles-sur-mer). It will be organized by the University of Caen in a joined meeting with the GP2A 26th Annual Medicinal Chemistry Conference.

